# Progress in Delivery of siRNA-Based Therapeutics Employing Nano-Vehicles for Treatment of Prostate Cancer

**DOI:** 10.3390/bioengineering7030091

**Published:** 2020-08-10

**Authors:** Milad Ashrafizadeh, Kiavash Hushmandi, Ebrahim Rahmani Moghadam, Vahideh Zarrin, Sharareh Hosseinzadeh Kashani, Saied Bokaie, Masoud Najafi, Shima Tavakol, Reza Mohammadinejad, Noushin Nabavi, Chia-Ling Hsieh, Atefeh Zarepour, Ehsan Nazarzadeh Zare, Ali Zarrabi, Pooyan Makvandi

**Affiliations:** 1 Department of Basic Science, Faculty of Veterinary Medicine, University of Tabriz, Tabriz 5166616471, Iran; dvm.milad73@yahoo.com; 2 Department of Food Hygiene and Quality Control, Division of Epidemiology & Zoonoses, Faculty of Veterinary Medicine, University of Tehran, Tehran 1419963114, Iran; houshmandi.kia7@ut.ac.ir (K.H.); sbokaie@ut.ac.ir (S.B.); 3 Department of Anatomical Sciences, School of Medicine, Student Research Committee, Shiraz University of Medical Sciences, Shiraz 7134814336, Iran; Ebrahimrahmani1374@gmail.com; 4 Laboratory for Stem Cell Research, Shiraz University of Medical Sciences, Shiraz 7134814336, Iran; zarrin.vahideh2075@gmail.com; 5 Department of Life Sciences, Islamic Azad University, North Tehran Branch, Tehran 1667914161, Iran; Sh.hz1989@gmail.com; 6 Radiology and Nuclear Medicine Department, School of Paramedical Sciences, Kermanshah University of Medical Sciences, Kermanshah 6715847141, Iran; najafi_ma@yahoo.com; 7 Cellular and Molecular Research Center, Iran University of Medical Sciences, Tehran 1449614525, Iran; shimal.tavakol@yahoo.com; 8 Pharmaceutics Research Center, Institute of Neuropharmacology, Kerman University of Medical Sciences, Kermaan 55425147, Iran; r.mohammadinejad87@gmail.com; 9 Research Services, University of Victoria, Victoria, BC V8W 2Y2, Canada; nabavinoushin@gmail.com; 10 Ph.D. Program for Translational Medicine, College of Medical Science and Technology, Taipei Medical University, Taipei City 110, Taiwan; chsieh2@tmu.edu.tw; 11 Department of Biotechnology, Faculty of Biological Science and Technology, University of Isfahan, Isfahan 8174673441, Iran; atefeh.zarepour@gmail.com; 12 School of Chemistry, Damghan University, Damghan 3671641167, Iran; ehsan.nazarzadehzare@gmail.com; 13 Sabanci University Nanotechnology Research and Application Center (SUNUM), Tuzla, Istanbul 34956, Turkey; 14 Center of Excellence for Functional Surfaces and Interfaces (EFSUN), Faculty of Engineering and Natural Sciences, Sabanci University, Tuzla, Istanbul 34956, Turkey; 15 Istituto Italiano di Tecnologia, Centre for Micro-BioRobotics, viale Rinaldo Piaggio 34, 56025 Pontedera, Pisa, Italy; 16 Chemistry Department, Faculty of Science, Shahid Chamran University of Ahvaz, Ahvaz 61537-53843, Iran

**Keywords:** small interfering RNA (siRNA), prostate cancer, gene therapy, nanoparticle, delivery systems, cancer therapy

## Abstract

Prostate cancer (PCa) accounts for a high number of deaths in males with no available curative treatments. Patients with PCa are commonly diagnosed in advanced stages due to the lack of symptoms in the early stages. Recently, the research focus was directed toward gene editing in cancer therapy. Small interfering RNA (siRNA) intervention is considered as a powerful tool for gene silencing (knockdown), enabling the suppression of oncogene factors in cancer. This strategy is applied to the treatment of various cancers including PCa. The siRNA can inhibit proliferation and invasion of PCa cells and is able to promote the anti-tumor activity of chemotherapeutic agents. However, the off-target effects of siRNA therapy remarkably reduce its efficacy in PCa therapy. To date, various carriers were designed to improve the delivery of siRNA and, among them, nanoparticles are of importance. Nanoparticles enable the targeted delivery of siRNAs and enhance their potential in the downregulation of target genes of interest. Additionally, nanoparticles can provide a platform for the co-delivery of siRNAs and anti-tumor drugs, resulting in decreased growth and migration of PCa cells. The efficacy, specificity, and delivery of siRNAs are comprehensively discussed in this review to direct further studies toward using siRNAs and their nanoscale-delivery systems in PCa therapy and perhaps other cancer types.

## 1. Introduction

Prostate cancer (PCa) is one of the leading causes of death in men worldwide and takes the second place for incidence and fifth place among cancer-related deaths in men [1]. Annually, 1.3 million new cases are diagnosed with PCa, out of which 359,000 cases result in death [2]. In the United States of America, approximately 174,650 new cases were diagnosed in 2019 alone [3,4]. PCa affects 30% of men over 50 years of age with only 10% of cases showing clinically significant symptoms [5]. Surgery, radiotherapy, chemotherapy, and hormone therapy are common therapeutic strategies in PCa therapy [6]. When PCa recurrence occurs or when patients with PCa are diagnosed with advanced stages, main therapy becomes androgen ablation using luteinizing hormone releasing hormone (LHRH) agonists and antagonists and/or anti-androgen receptors (ARs) [7,8]. It is noteworthy that patients with PCa typically develop metastatic castration-resistant prostate cancer (mCRPC) [9]. Although patients with mCRPC can be treated with chemotherapeutic agents such as taxanes, immunotherapy, radiotherapy, or hormone therapy, these treatments can only improve the survival rate of patients by 2–4 months [10,11]. In addition to the aforementioned issues, PCa cells develop resistance to radiotherapy and chemotherapy, causing a clinical relapse [12,13,14,15].

This calls for extensive research into PCa to shed light on the number of strategies that can target PCa more effectively. The positive aspects are that the prostate is a nonvital organ and amenable to the use of tissue-specific antigens. Due to the fact that PCa is small in size and not very deep in the body, it provides excellent antibody access and penetration. mCRPC clinically manifests in lymph nodes and bones with high levels of circulating antibodies, making its detection easy. Finally, the prostate-specific antigen (PSA) serum marker allows the early detection of metastatic PCa [16]. Since PCa lacks clinical symptoms in early stages, its definitive detection depends on prostate biopsy, alterations in PSA levels, and/or digital rectal examinations (DRE) [17]. Research so far shows that cell-surface proteins, glycoproteins, receptors, enzymes, and peptides are considered as targets in PCa therapy [18,19,20].

Understanding molecular pathways involved in PCa malignancy is key to effective treatment and targeting. Studies published in recent years focused on revealing identified molecular signaling pathways. The common theme in these studies is that tumorigenesis emanates from an alteration in the normal expression of onco-suppressor or oncogene factors [21,22]. Regardless of how we deal with gene expression changes, expanding our knowledge of upstream and downstream genetic mediators can pave the way in cancer therapy [23,24]. Effective cancer therapy not only depends on finding the specific biomarkers, but also understanding intermediary regulators [25,26,27]. Such understanding can facilitate the process of cancer therapy and finding novel cures. As evidenced by most current research findings, PCa cells show malignant properties at advanced stages and metastasize. Accumulating data demonstrate that the Wnt signaling pathway partially participates in metastasis. In eradication of metastatic PCa, Wnt5A ligand or its downstream targets such as Frizzled (*FZD*) receptors (*FZD4* and *FZD8*) and c-Jun N-terminal kinase (JNK) pathway may be targeted [28]. Notably, there are factors that can function as upstream mediators of Wnt in PCa. Activation of keratin 5 (KRT5) can inhibit the Wnt signaling pathway, resulting in inhibition of growth and invasion of PCa cells. The *KRT5* gene is a downstream target of onco-suppressor microRNA (miR)-601, suggesting that the miR-601/KRT5/Wnt axis can be targeted in future studies for inhibition of PCa [29]. More importantly, miRs can be considered as downstream targets of long non-coding RNAs (lncRNAs) in PCa. For instance, lncRNA LINC00665 and PROX1-AS1 can respectively target miR-1224-5p and miR-647 in enhancing the malignant tendencies of PCa cells [30,31]. These studies are in line with the fact that dynamic and complex molecular pathways are involved in Pca malignancy [32]. Some of them are oncogene factors participating in increasing progression and malignancy of cancer cells, while others are onco-suppressor factors that can be regulated in the treatment of PCa [33,34,35,36]. The result of revealing the role of these pathways is an opportunity for the development of anti-tumor compounds in PCa therapy as confirmed by onco-suppressor studies [37]. For instance, ursolic acid can limit the progression and proliferation of PCa cells via upregulation of onco-suppressor gene *PTEN*, while quercetin suppresses the malignancy of PCa cells through downregulation of oncogene *PI3K/Akt* [38,39]. Despite these developments, PCa treatment remains increasingly challenging for clinicians, suggesting the need for further research. In the current review, we discusse one of the major efforts in PCa treatment using small interfering RNA (siRNA) tools. We then expand our discussion toward using nanoparticles for targeted delivery of siRNA in PCa therapy and suggest the exploration of their potential in other cancer types.

## 2. siRNA Structure and Function: A Brief Overview

**Figure 1:**
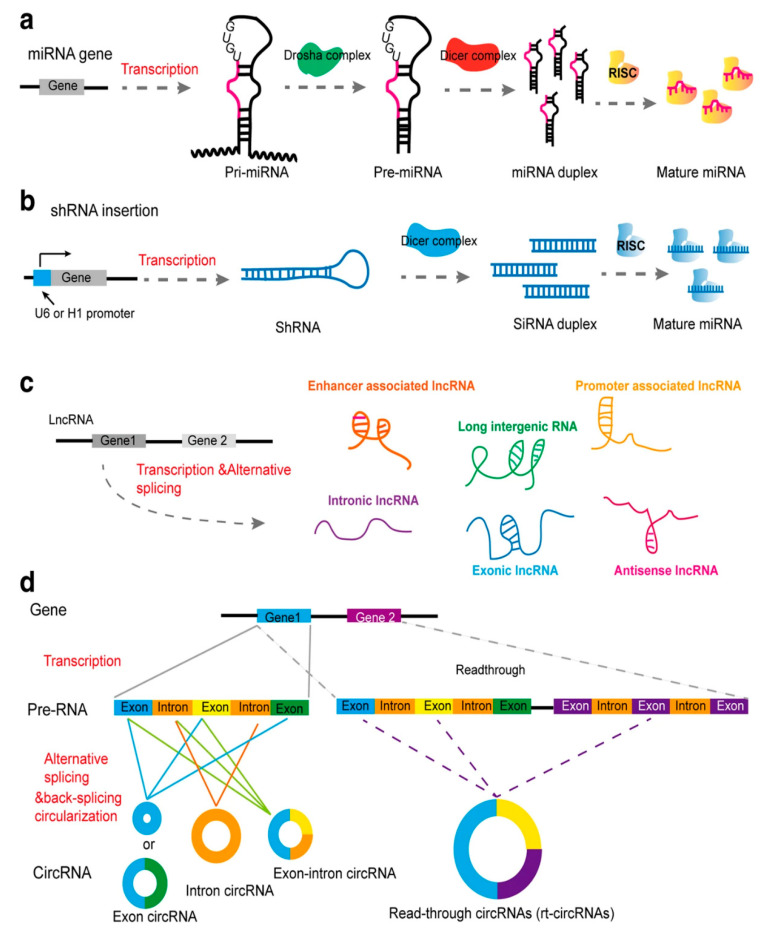
The biogenesis of some types of non-coding RNA. (**a**) Biogenesis of microRNA with at least one hairpin. (**b**) The biogenesis of small interfering RNA (siRNA) derived from short hairpin RNA (shRNA). (**c**) Biogenesis of long non-coding RNAs (LncRNAs) transcribed in the genome. (**d**) Biogenesis of circular RNA (circRNA) mostly derived from pre-messenger RNAs (mRNAs). miRNA, micro RNA; pri-miRNA, primary micro RNA; pre-miRNA, precursor-miRNA; shRNA, small hairpin RNA; siRNA, small interfering RNA; LncRNA, long non-coding RNA; CircRNA, circular RNA [62].

Over the past decades, we witnessed a close relationship between the field of molecular biology and medicine, with molecular biologists having interests in developing novel strategies in the treatment, prevention, and management of cancer (Figure 1) [40,41,42,43,44,45,46,47,48,49,50]. One of the most important discoveries made by molecular biologists is the introduction of RNA interference (RNAi), enabling the targeting of certain genes in the treatment of cancer [51]. Among the various kinds of RNAi tools, miRs and siRNAs are of importance in cancer therapy [52]. There are a number of differences between miRs and siRNAs. The first difference is that miRs are formed endogenously from non-coding RNAs, while siRNAs are produced by exogenous long double-stranded RNAs (dsRNAs) [53,54]. The transportation of miRs during their biogenesis on the route of the nucleus to the cytoplasm is performed via importin 8 (IPO-8). Using siRNA-IPO8 enables us to suppress miR activation via inhibiting its translocation [55]. It is worth mentioning that a characteristic cellular machinery is involved in the formation of siRNAs from exogenous short hairpin RNA precursors. These kinds of siRNAs are able to effectively silence target genes [56]. Structurally, an siRNA is a double-stranded RNA molecule with 21–23 nucleotides in each strand [57]. After binding to the RNA-induced silencing complex (RISC) in the cytoplasm, the sense strand of siRNA undergoes cleavage and ejection, while the antisense strand of siRNA targets the complimentary messenger RNA (mRNA) thermodynamically. From this point, two distinct events occur. The partial hybridization of antisense strand of siRNA with the target mRNA leads to inhibition of translation, while perfect complementary hybridization results in mRNA degradation [58,59,60,61]. This demonstrates that siRNA exerts an inhibitory effect on the expression of the target gene.

Due to the capability of siRNA in reducing the expression of target genes, studies focused on using siRNA in the downregulation of oncogene pathways in cancer therapy. As an example, pyruvate kinase is a rate-limiting enzyme participating in glycolysis for the conversion of phosphoenopyruvate (PEP) and ADP to pyruvate and ATP. Four isoforms of pyruvate kinase exist and, among them, PKM2 is of interest in effective cancer therapy because of its critical role in enhancing the proliferation and invasion of cancer cells [63,64,65,66]. Recently (2020), an effort was made to knock down PKM2 using siRNA. The results are in agreement with the reduced growth of cancer cells due to downward regulation of oncogene factor *PKM2* [67]. The nuclear factor kappa B (NF-κB) is another oncogene signaling pathway involved in the growth and invasion of cancer cells [68]. It appears that downregulation of NF-κB using siRNA can pave the way to the eradication of melanoma cancer cells, while also suppressing their metastasis [69]. In addition to the NF-κB signaling pathway, Aurora-A can be targeted in restricting the metastasis of cancer cells. The inhibition of Aurora-A using siRNA is correlated with a decrease in migration and invasion of cancer cells [70]. B-cell lymphoma 2 (Bcl-2) is a key protein of apoptosis with pro-survival roles. The upregulation of Bcl-2 in cancer cells occurs via the inhibition of apoptosis [23]. Silencing of Bcl-2 using siRNA induces apoptosis in cancer cells and diminishes their proliferation [71]. Thus, we are increasingly witnessing the potential of siRNA in cancer therapy and how siRNA treatment can be used as a tool to accelerate our pace in the treatment and eradication of cancer(s) [72]. A study was conducted on using siRNA tools in the treatment of cancer patients. In this study, CALAA-01 was administered to 24 patients. CALAA-01 is a polymer-based nanoparticle having siRNA. It was found that elimination of CALAA-01 from the body depends on weight. Notably, it was well tolerated in humans, and there was no associated toxicity [73]. This study confirmed that (1) siRNA and its encapsulation by nanoparticles can be applied in clinical trials, (2) nanoscale-mediated siRNA delivery is biocompatible, and (3) animal models can predict the behavior of siRNA-based technologies in human. In the next section, we specifically discuss the efficacy of siRNAs in the treatment of PCa and in improving the prognosis of patients with this disease.

## 3. siRNA Targets Signaling Pathways: Focus on PCa Therapy

**Figure 2:**
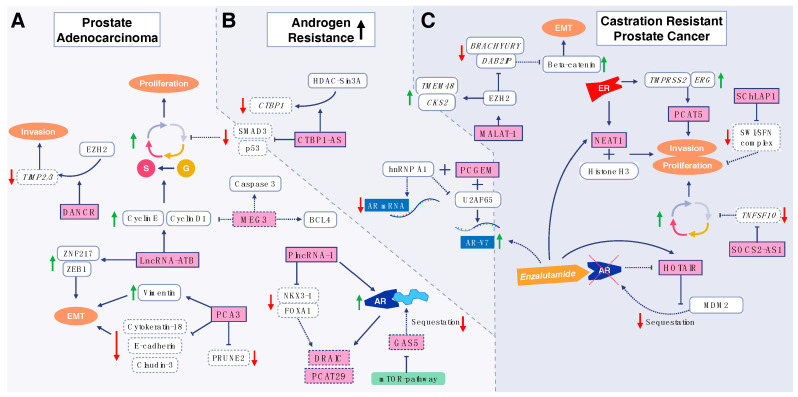
Molecular functions of lncRNAs at various steps of prostate cancer (PCa): (**A**) prostate adenocarcinoma; (**B**) castration resistance; (**C**) castration-resistant state. LncRNAs are colored in red, angular shaped boxes. Genes and proteins are colored in white boxes with blunt edges. Reprinted with permission from Reference [93].

Apart from gene expression dysregulation, mutations in genes can also result in the development and progression of PCa. In this way, siRNA can be beneficial via targeting signaling pathways involved in the malignancy of PCa cells. As a transcription factor, special AT-rich sequence-binding protein 1 (SATB1) functions in histone modification regulation and modulation of gene expression (Figure 2) [74]. A variety of studies demonstrated that SATB1 undergoes upregulation in various cancers, and it is correlated with migration, proliferation, and unfavorable prognosis [75,76]. Thus, targeting SATB1 is of importance in PCa therapy. It was shown that downregulation of SATB1 using siRNA can pave the way for a reduction in growth, proliferation, and metastasis of PCa cells [77]. The siRNA-mediated Bcl-xL downregulation potentiates the inhibitory effect on the malignancy and growth of PCa cells [78]. Another example of successful siRNA treatment is the tripartite motif-containing protein 24 (TRIM24), a carcinogenesis factor capable of enhancing progression and viability of different cancers [79,80]. The strategy is based on suppressing TRIM24 in cancer therapy [81]. The treatment is based on in vitro and in vivo experiments showing that TRIM24-siRNA is effective in the eradication of PCa cells. This is because, upon downregulation of TRIM24, a decrease is observed in the proliferation, colony formation, and invasion of PCa cells [82]. Protein phosphatase 2A (CIP2A) is another oncogene factor participating in the malignancy of cancer cells and enhancing their growth and proliferation [83,84]. It was demonstrated that PCa cells elevate the expression of CIP2A to ensure their proliferation and malignancy [85,86]. It is worth mentioning that the overexpression of CIP2A mediates chemoresistance [87,88]. Thus, suppressing CIP2A expression not only reduces the proliferation of cancer cells, but also sensitizes them to chemotherapy. It was in fact shown that siRNA-mediated CIP2A knockdown diminishes the resistance of PCa cells to docetaxel-induced apoptosis [89]. With respect to the uncontrolled growth and proliferation of PCa cells, the identification of biomarkers involved in proliferation is key in targeting them for therapy. Poly(ADP-ribose) polymerase-1 (PARP1) attaches to DNA strand breaks to form long branched polymers of poly(ADP-ribose) using NAD^+^. *PARP1* plays a significant role in preserving genome stability and performing DNA repair [90,91], ensuring the growth and proliferation of cancer cells. The downregulation of PARP1 using siRNA dually affects both the metastasis and the proliferation of PCa cells. In suppressing the invasion of cancer cells, siRNA-mediated PARP1 inhibition leads to a reduction in epithelial-to-mesenchymal transition (EMT) via upregulation of E-cadherin and downregulation of vimentin. In suppressing the growth of PCa cells, downregulation of PARP1 results in inhibition of *PI3K/Akt* genes [92]. These studies highlight the fact that using siRNA is advantageous in suppressing PCa malignancy via negatively targeting both the migration and the proliferation of cancer cells.

In addition to the inhibition of chemoresistance, siRNA can be applied to enhancing the anti-tumor activity of chemotherapeutic agents. Hypoxia-inducible factor-1 alpha (HIF-1α) is a cancer-related transcription factor capable of the stimulation of enzymes involved in glycolysis. Accumulating data demonstrate that HIF-1α enhances the metastasis and proliferation of cancer cells. Furthermore, HIF-1α can trigger the chemoresistance of tumor cells [94,95,96,97]. This resulted in much attention toward the inhibition of HIF-1α expression in suppressing chemoresistance, while elevating the anti-tumor activity of chemotherapeutic agents. In PCa cells, siRNA-mediated HIF-1α downregulation results in a reduction in glycolysis and mitochondrial oxidative phosphorylation, paving the way for the enhanced production of reactive oxygen species (ROS) and the stimulation of cell death. Hence, siRNA can be beneficial in enhancing the sensitivity of PCa cells to cisplatin chemotherapy [98].

Another usage of siRNAs in cancer therapy is through leveraging the molecular pathways that are involved in angiogenesis. For instance, the c-Jun *N*-terminal kinase (JNK) pathway, a member of the mitogen-activated protein kinase (MAPK), results in a reduction in DNA damage [99,100], and the administration of cisplatin is corelated with stimulation of the JNK signaling pathway. It was shown that siRNA-JNK can enhance the sensitivity of PCa cells to cisplatin chemotherapy via the induction of DNA damage [101]. For instance, endothelial cell-specific molecule-1 (ESM-1) is an oncogene factor that is upregulated in various cancers [102]. ESM-1 is able to induce angiogenesis by functioning as an upstream mediator, targeting vascular endothelial growth factor (VEGF) [103,104]. Additionally, CXC chemokines can trigger angiogenesis [105]. The downregulation of ESM-1 via siRNA diminishes the expression of *CXCL3*, leading to a decrease in the migration and metastasis of PCa cells by suppressing angiogenesis [106].

Another example is Sal-like 4 (SALL4), an oncogene factor with stimulatory impacts on the proliferation and metastasis of cancer cells [107,108]. Decreasing the expression of *SALL4* using siRNA stimulates apoptotic cell death in PCa cells via upward regulation of pro-apoptotic factor Bax and downward regulation of anti-apoptotic factor Bcl-2 [109].

**Figure 3:**
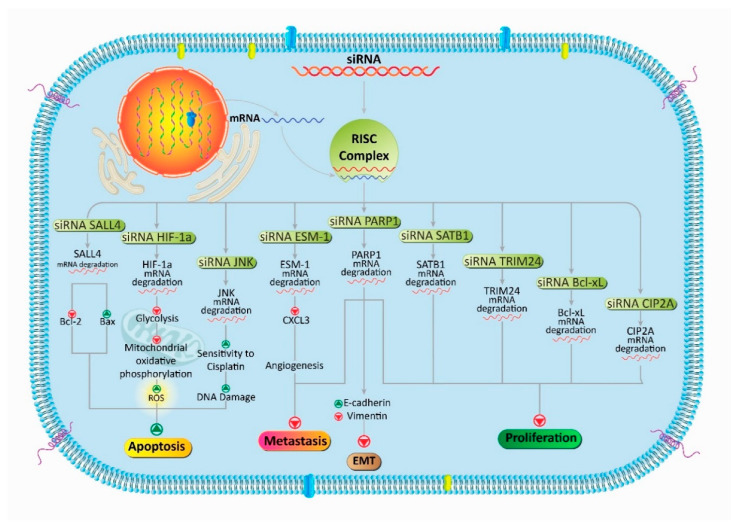
A schematic representation of using siRNA in PCa therapy. Oncogene molecular pathways that are involved in proliferation and migration such as PARP/EMT, CIP2A, TRIM24, and so on can be affected using siRNA. In addition, siRNA can be used in the induction of apoptosis (Bcl-2 downregulation and Bax upregulation) and in suppressing the glycolysis (metabolism) of PCa cells.

Taking everything into account, these studies are in line with the fact that dynamic and complicated molecular signaling pathways contribute to the malignant behavior of PCa cells [110,111]. The first step is the recognition of these identified molecular pathways and the additional research being undertaken to identify more molecular pathways involved in PCa malignancy [112,113,114]. The next step is designing specific and efficacious siRNAs for targeting the identified molecular signaling pathways for PCa therapy (Figure 3) [114,115]. Table 1 summarizes the efforts related to the knockdown of oncogene molecular pathways that may be considered for PCa therapy.

## 4. The Dark Side of siRNA Delivery System: Challenges and Opportunities

Although siRNAs show excellent efficiency in cancer therapy, there are still drawbacks to this tool. Reaching the site of cancer in deep tissues while still maintaining their integrity is one challenge. Nuclease activity can degrade siRNAs and reduce their efficiency in targeting genes. Furthermore, siRNAs have non-specific off-target side effects that may induce immune responses [134]. It was suggested that certain sequences of siRNA can target Toll-like receptors (TLRs) such as TLR-7, TLR-8, and TLR-9, as well as RIG1 [135,136], leading to immune response activation. Therefore, efforts were made to modify siRNAs such as changing their backbone to inhibit immune responses and nuclease degradation. It is said that substitutions on the 2′ carbon of ribose provides protection of siRNA against degradation. Notably, modification of the 2′ *O*-methyl suppresses siRNA-mediated immune stimulation [137]. The inverted abasic ribose at the end of the siRNA strand inhibits nuclease degradation [137]. Abnormal structures at the end of each strand of the siRNA lead to challenges in the incorporation of siRNA into RISC complexes. Modification of this structure overcomes the issues in incorporating siRNAs into the RISC [138,139,140]. Even though these modifications greatly helped us in improving the efficiency of siRNA in cancer therapy and the modulation of gene expression, there is still need for further research.

In the case of PCa, same problems are observed. Firstly, siRNA should circulate in the bloodstream and, in this way, it may undergo enzymatic degradation. Then, it should endure the mild acidic pH of the tumor microenvironment and be capable of selectively targeting PCa cells. However, siRNA possesses off-target features that should be considered during PCa therapy. Thus, protection against degradation and internalization are challenges for the siRNA system in PCa cells, which can be solved using nanoscale delivery systems [141].

In brief, the strategy of using nanoparticles for the delivery of siRNA significantly improved the potential of siRNA in cancer therapy [142].These nanostructures were in fact applied in clinical trials for the delivery of siRNA with high efficiency [143,144]. To date, various nanoparticles such as polymeric nanoparticles, lipid nanoparticles, carbon nanotubes, and gold nanoparticles were designed for the delivery of siRNA [51,88,145,146,147,148]. These nano-vehicles provide protection for the siRNA against degradation and a reduction of the off-target effects via delivery to targeted sites [149,150]. In the next section, we comprehensively discuss the efficiency of different kinds of nanoparticles for the delivery of siRNA with potential in PCa therapy.

## 5. Nano-Vehicles

**Figure 4:**
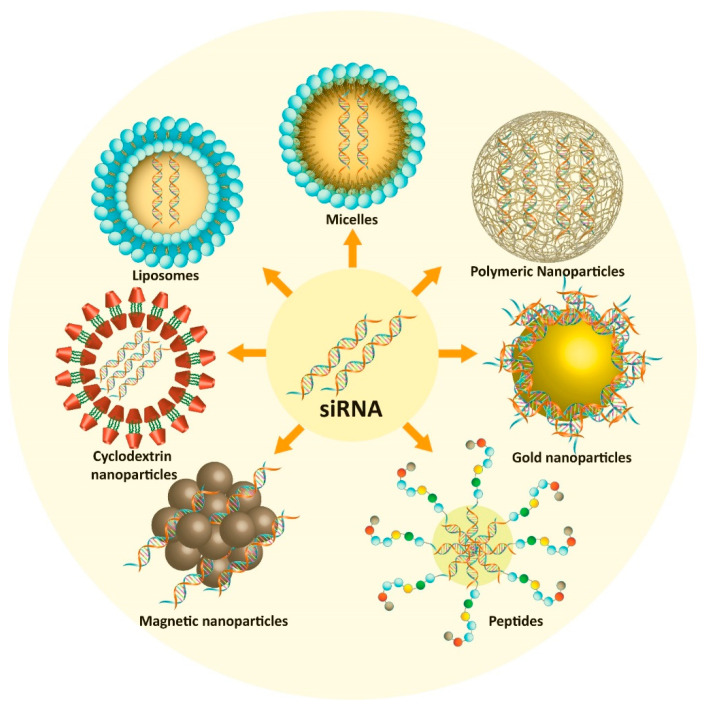
Nanostrategies used for siRNA delivery in effective prostate cancer therapy.

In the previous sections, we demonstrated that siRNAs represent an emerging strategy for cancer therapy. However, one of the difficulties is the limitation of targeted delivery to the site of cancer, including PCa [151,152,153,154,155]. To date, various carriers were designed for the delivery of siRNA for PCa, such as polymeric nanoparticles, lipid nanoparticles, nanobubbles, and cyclodextrins [156,157]. These vehicles are able to deliver siRNAs into the tumor site and reduce the proliferation and malignancy of PCa cells [73,158,159]. Moreover, they provide a platform for the co-delivery of siRNA and other chemotherapeutic agents that may be beneficial for effective PCa therapy [160,161]. These vehicles are discussed in this section and summarized in Table 2. Figure 4 shows the different nanocarriers employed for the delivery of siRNA in prostate cancer therapy.

### 5.1. Polymeric Nanoparticles

Dendrimers are members of dendritic polymers with a variety of features such as well-defined and controlled structures, monodispersity, and multivalent properties [162,163,164,165]. Despite having these properties, amino acids can be used as branching units that form peptide dendrimers and improve their adhesive properties. It was demonstrated that peptide dendrimers have high biocompatibility and are resistant to proteolytic digestion [166,167,168]. This resulted in the application of peptide dendrimers for the delivery of drug and gene materials [169]. An effort was made for delivery of HSP27-siRNA using peptide dendrimers in the treatment of PCa. The peptide dendrimers can protect siRNAs against enzymatic degradation, leading to their enhanced efficacy in gene silencing. The increased potential of siRNAs by peptide dendrimers is not only due to their protection against enzymatic degradation, but also to the fact that siRNA-loaded peptide dendrimers demonstrate high cellular uptake and release siRNA in an endosome-release manner. The siRNA-loaded peptide dendrimers are capable of effectively silencing the *HSP27* gene, an oncogene involved in the survival and proliferation of PCa cells, with more than 60% leading to high anti-tumor activity [170]. Although polymeric nanoparticles have great potential in gene delivery, surface modification can enhance their benefits in cancer therapy.

Another example is the use of arginine–glycine–aspartic acid (RGD) for specific targeting of PCa cells, as cancer cells are abundant in neovascular vessels and avb3 integrin is upregulated in these tumors [171,172]. Surface modification of polymeric nanoparticles with RGD enhances their efficacy in targeting PCa cells. The stability of RGD-modified polymeric nanoparticles leads to effective targeting. In this example, siRNA with an entrapment efficiency of about 83.8% ± 5.71% led to downregulation of *GRP78*, an oncogene that suppresses the malignant behavior of PCa cells, such that the expression of this gene was less than 34% while free siRNA showed gene expression of about 83% [173].

Multifunctional polymeric nanoparticles can be considered as ideal candidates in PCa therapy. For instance, pH-responsive nanoparticles can release drugs or genes at the mildly acidic pH of the tumor microenvironment (pH 6 to 6.5). The immediate disassembly of multifunctional nanoparticles at this pH provides the targeted delivery of drugs or genes at tumor sites [174,175]. Notably, the disassembled components can penetrate the endosomal membrane of cancer cells, releasing the drugs or genes into the cytoplasm [176,177]. Multifunctional polymeric nanoparticles are used for the delivery of siRNA-prohibitin-1 (PHB1) in PCa cells. PHB1 is a 32-kDa protein capable of regulating various cellular pathways such as apoptosis, proliferation, and transcription [178,179]. The expression of PHB1 shows an increase in cancer cells [180,181], making it a suitable target in cancer therapy. In order to enhance the capability of these nanoparticles in targeting PCa cells, multifunctional polymeric nanoparticles were modified by ACUPA, which targets and identifies the prostate-specific membrane antigen (PSMA). The cytoplasmic delivery of siRNA-PHB1 with these nanoparticles (with different entrapment efficiencies from 51.8–92.1%) led to downregulation of this oncogene to about 60–90%, as well as a decrease in the malignancy of PCa cells [182].

### 5.2. Lipid Nanostructures

**Figure 5:**
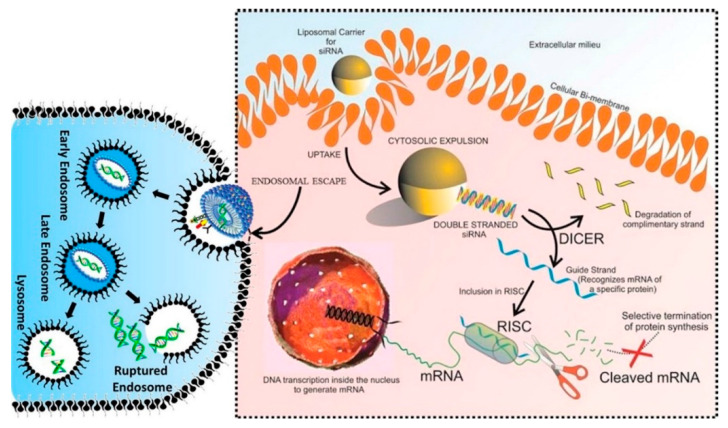
The application of siRNA encapsulated into a liposome for cancer therapy. RISC, RNA-induced silencing complex. Reprinted with permission from Reference [183].

**Figure 6:**
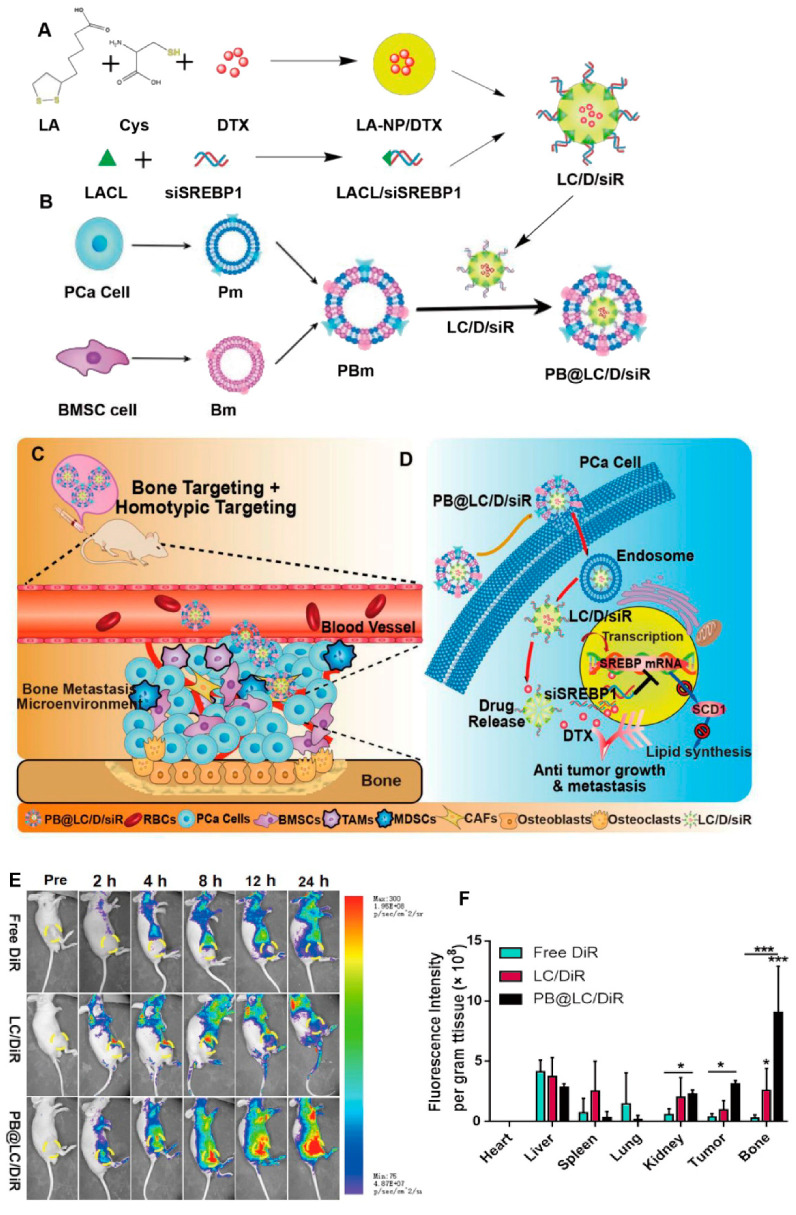
(**A**) The preparation of co-loading nanoparticles LC/D/siR. (**B**) The fusion and coating of PBm. (**C**) The schematic illustration of PB@LC/D/siR targeting the microenvironment of BmCRPC based on the fundamental bone homing and homotypic targeting ability of PBm. (**D**) The mechanism of PB@LC/D/siR. (**E**) The representative small animal living images of each group of the BmCRPC-bearing mice at 0–24 h post injection (yellow circle: tumor area). (**F**) The qualified distribution in major organs of each group. DTX, docetaxel; LA, lipoic acid. Reprinted with permission from Reference [190].

Micelles are core–shell nanoparticles produced by spontaneous self-assembly of individual amphiphilic (hydrophobic/hydrophilic) molecules in water or other aqueous solutions [183]. Micellar nanoparticles can protect hydrophobic drugs and genes in their micelle core and, because of their small size (less than 100 nm), they are extensively applied to gene or drug delivery (Figure 5, [184]) [185,186]. Notably, micelles were used for the delivery of siRNA in cancer therapy with success [187,188]. For instance, an experiment used micelles for delivery of siRNA-SREBP1 to PCa cells. SREBP1 (sterol regulatory element-binding protein) is an oncogene in PCa, and its interaction with PKD3 enhances the proliferation of PCa cells [189]. It was observed that micelles can successfully co-deliver docetaxel and siRNA-SREBP1 to Pca cells. Downregulation of SREBP1 led to a diminution in the invasion, metastasis, and growth of PCa cells, while sensitizing them to docetaxel chemotherapy such that cells exposed to both siRNA and docetaxel showed 4.9-fold toxicity in comparison to cells exposed to docetaxel alone. Protection of siRNA-SREBP1 against degradation increased its efficacy (Figure 6) [190]. Notably, intravenous (i.v.) administration of lipid nanoparticles containing siRNA-AR suppressed PCa cell viability and reduced serum levels of PSA to about 40% in comparison to a control mouse model [191]. The inhibitory effect on the malignancy of PCa cells was further improved by blocking PSMA and extinguishing the expression of *AR*, leading to complete AR silencing and about a 50% reduction in the growth and malignancy of cancer cells [151].

**Figure 7:**
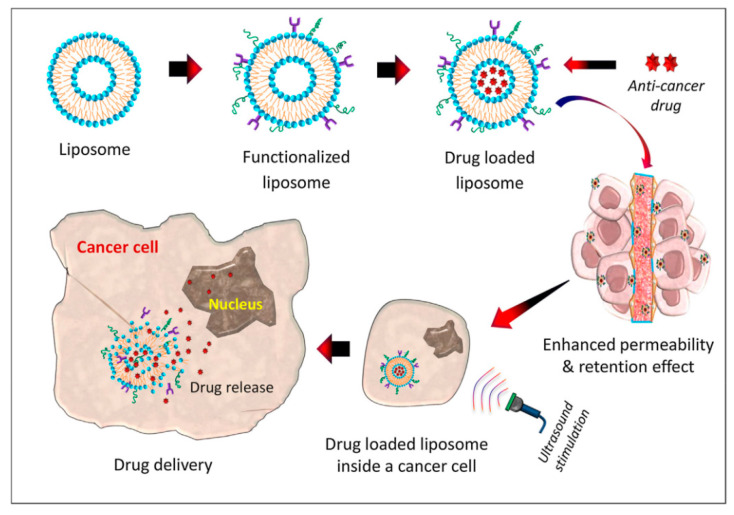
Schematic fabrication and utilization of smart liposome for cancer therapy. Reprinted with permission from Reference [192].

In addition to micelles, there exist other types of lipid-based nanoparticles used as carriers for drug and gene delivery in cancer treatment, such as liposomes, solid lipid nanoparticles, noisomes, etc. Liposomes are bilayer vesicles consisting of different types of phospholipids and cholesterol, which are arranged together so that they can be used as a carrier for both hydrophobic and hydrophilic components. They can also be engineered with several functionalizing agents that prepare them for use as targeted smart delivery systems (Figure 7) [192].

**Figure 8:**
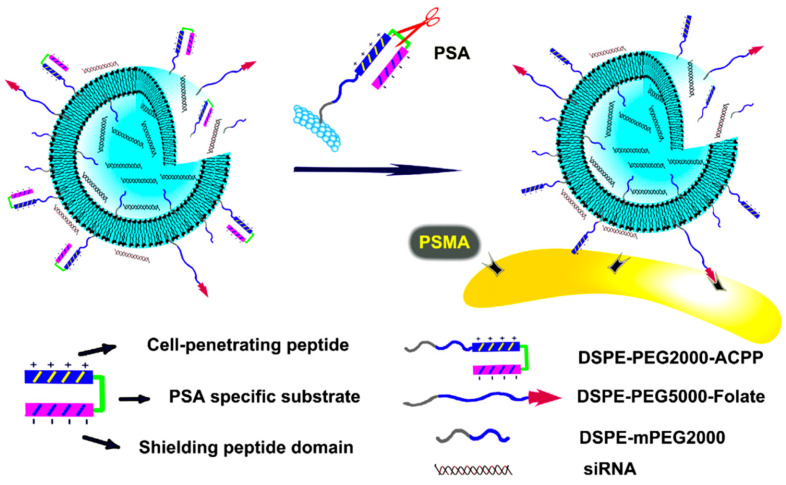
Smart multifunctional liposome-siRNA containing folate and activatable cell-penetrating peptide (ACPP) targeting moieties against prostate-specific membrane antigen (PSMA) and glycosaminoglycans on the cell surface. Abbreviations; DSPE-mPEG2000: 1,2-distearoyl-*sn*-glycero-3-phosphoethanolamine-*N*-methoxy (polyethylene glycol), DSPE-PEG2000- ACPP: 1,2-distearoyl-*sn*-glycero-3-phosphoethanolamine-*N*-maleimide(polyethylene glycol)–activatable cell-penetrating peptide, PSA: prostate-specific antigen, PSMA: prostate-specific membrane antigen. Reprinted with permission from Reference [193].

There are several studies in which functionalized liposomes were used for treating prostate cancer via applying siRNA. In one study, a type of multifunctional liposome was prepared via applying stealth liposomes (liposomes coated with polyethylene glycol) which were used for the encapsulation of siRNA and protecting it from lysosomal digestion. These liposomes were functionalized by two types of components: folate as the targeting agent, which showed a high affinity for the attachment to the prostate-specific membrane antigen (PSMA), and a prostate-specific antigen (PSA)-sensitive peptide. The PSA-sensitive peptide consisted of three parts including the cell penetration segment (polyarginine), which was a type of cell-penetrating peptide (CPP) with positive charge that enhanced the intracellular delivery of liposomes, the PSA-sensitive cleavable peptide (HSSKYQ), which was responsible for the amount of PSA and donated the smart ability to this type of liposome, and the polyanionic inhibitory peptide (DGGDGGDGGDGG), which was a negatively charged domain used as shielding to protect the positively charged domain. The performance of this type of liposome was dependent on the amount of PSA, which is found at a high level in the microenvironment of prostate cancer. In the extracellular microenvironment of prostate cancer, PSA acted as an enzyme and cleaved the PSA-sensitive peptide, which led to the appearance of the CPP domain that promoted the cellular uptake of liposomes (Figure 8). This liposome showed a significant effect on cell uptake, increasing apoptosis in prostatic cancer cells via preserving the siRNA that reduced polo-like kinase 1 (PLK-1) expression by 22–75% (based on the type of synthesized liposome) [193].

**Figure 9:**
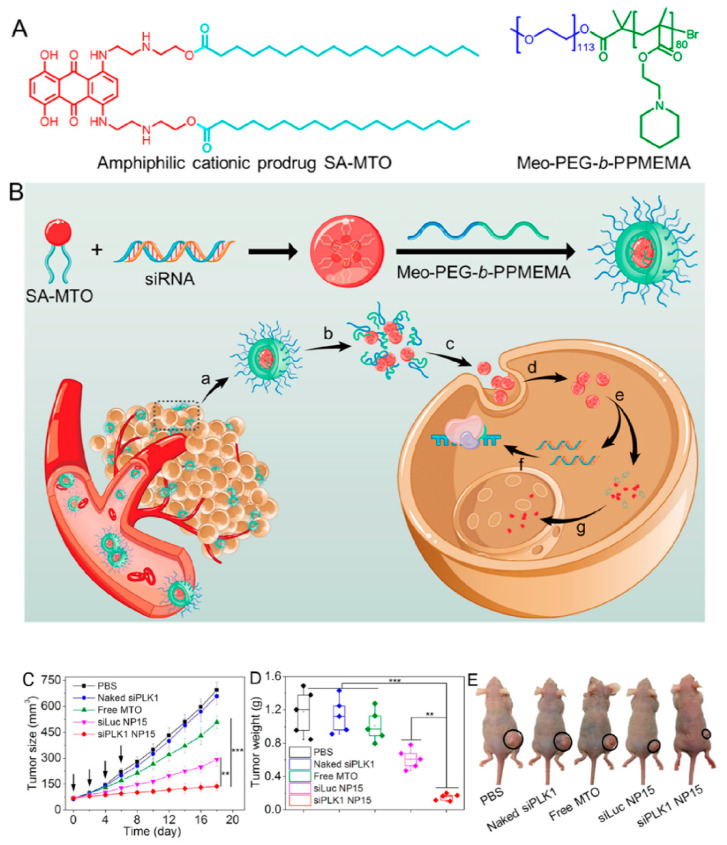
(**A**) Chemical structure of the amphiphilic cationic prodrug siRNA amphiphilic cationic mitoxantrone (SA-MTO) and TME pH-responsive polymer methoxyl-poly (ethylene glycol)-b-poly (2-(pentamethyleneimino) ethyl methacrylate (Meo-PEG-b-PPMEMA). (**B**) Schematic illustration of the TME pH-responsive polymer–prodrug hybrid nanoplatform for multistage siRNA delivery and combination cancer therapy. Tumor size (**C**) and weight (**D**) of the MDA-MB-231 xenograft tumor-bearing nude mice treated with phosphate-buffered saline (PBS), naked siPKK1, free MTO, and siLuc- and siPLK1-loaded NP15. (**E**) Representative photograph of the MDA-MB-231 xenograft tumor-bearing nude mice in each group at day 18. Meo-PEG-b-PPMEMA, methoxyl-poly (ethylene glycol)-b-poly (2-(pentamethyleneimino) ethyl methacrylate); SA-MTO, siRNA amphiphilic cationic mitoxantrone. Reprinted with permission from Reference [194].

Using prodrugs is a promising approach to enhance the selectivity and efficacy of chemotherapeutic drugs. Having this in mind, an amphiphilic cationic prodrug based on lipids was employed to load RNA therapeutics for co-delivery (Figure 9) [194]. The amphiphilic lipids formed nanoparticles in aqueous conditions and simultaneously encapsulated siRNA with an entrapment efficiency of about 35.1–68.9% (for different nanoparticles). Subsequently, the surface of the nanosized particles was decorated with polymers to enhance the hydrophilicity of the nanohybrid particles which, accordingly, prolonged the blood circulation and tumor accumulation. In addition, the polymer turned the particles into stimuli-responsive vehicles to respond to pH as a trigger. The findings showed that esterase (as overexpressed in the tumor microenvironment) led to cleavage of the prodrug, allowing the siRNA and anticancer drug to be efficiently liberated in the cytoplasm. These types of nanocarriers showed about 70% knockdown in the expression of PLK1 [194].

### 5.3. Peptides

Over the past few decades, we witnessed special attention toward peptides for their use as platforms for the delivery of genes and drugs. Peptides have a number of beneficial features including biocompatibility, biodegradability, minimal toxicity, and ease of synthesis [195,196], making them suitable options for the delivery of genes and drugs. To date, different peptides were designed for delivery, and the findings were satisfactory [197,198]. Notably, the potential of peptides in delivery can be improved by using a combination of phospholipids (lipoplex) and polymers (polyplex), which results in an improvement in the transfection efficiency of peptides [199,200,201,202]. In one study, hybrid peptides/phospholipids were used for delivery of siRNA-EGFP in PCa cells. Surface modification of these peptides using gastrin-releasing peptide receptor (GRPR) enhanced their cellular uptake through endocytosis. They had superior biocompatibility and delivered siRNA into PCa cells, which led to effective downregulation of EGFP (between 50% and 10% for different formulations) [203]. This study demonstrated that peptides are ideal candidates in siRNA delivery for reducing the viability of PCa cells, and their surface modification by receptors can improve their proficiency in cancer therapy.

Cell division cycle-associated protein 1 (CDCA1) is an element of the kinetochore complex that is important for the stability of the kinetochore and microtubule [204]. CDCA1 plays a considerable role in mitosis. The silencing of CDCA1 inhibits kinetochore–microtubule attachment, leading to death of mitotic cells [205]. It was reported that CDCA1 is upregulated in various cancers [206,207,208,209], and its downregulation is implicated in cancer therapy. In PCa cells, the cytoplasmic release of siRNA-CDCA1 via peptides led to inhibition of CDCA1 and stimulation of apoptotic cell death by about four-fold. An in vivo experiment also revealed that siRNA-CDCA1-loaded peptide diminished the tumor growth and volume, suggesting their efficacy and promise [210].

**Figure 10:**
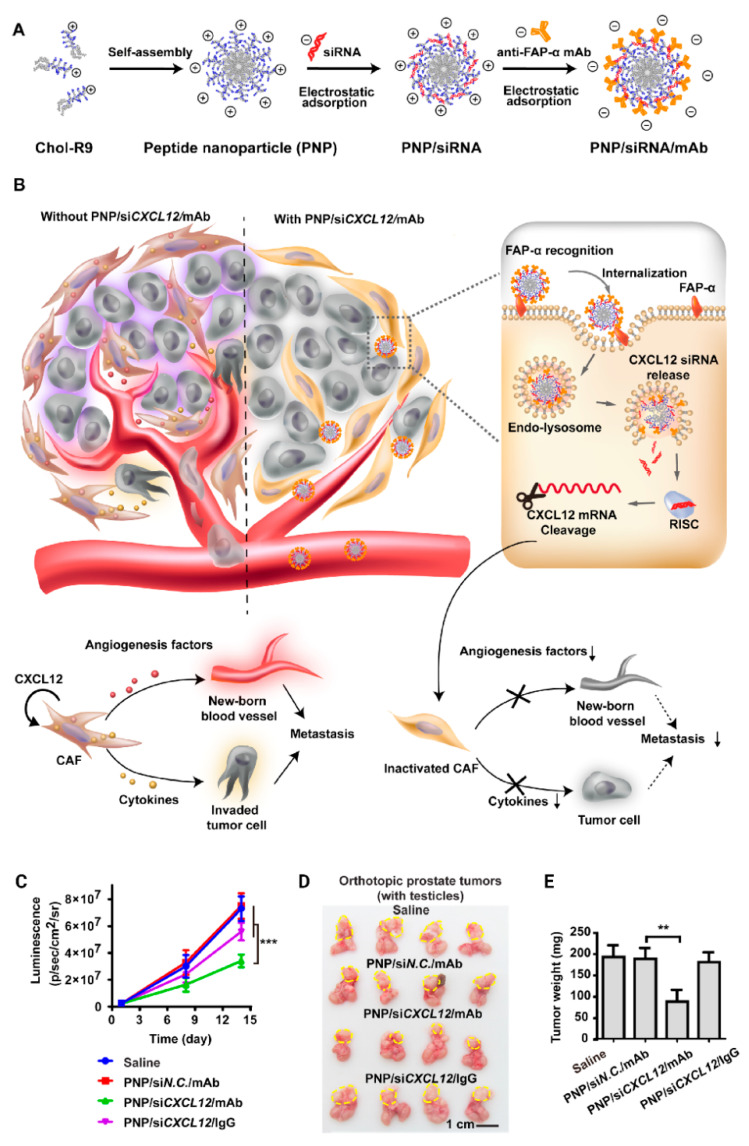
(**A**) Construction of the peptide nanoparticle (PNP)/siRNA/mouse antibody (mAb) nanosystem through a self-assembly process. (**B**) Proposed mechanism of PNP/siRNA-C–X–C motif chemokine ligand 12 (CXCL12)/mAb-mediated metastasis inhibition and cell-penetrating peptide (CPP)-mediated transfection of CXCL12 siRNA in cancer-associated fibroblasts (CAFs). (**C**) Tumor progression curves determined by quantification analysis of the in vivo bioluminescence signal. (**D**) Images of prostate tumors with testicles. Yellow dashed lines represent the locations of the primary tumor. (**E**) Weight of isolated tumors (without prostate and testicles) in each group. CPP, cell-penetrating peptide; CXCL12, C–X–C motif chemokine ligand 12; mAb, mouse antibody; PNP, peptide nanoparticle. Reprinted with permission from Reference [211].

Self-assembly is a promising approach to prepare nanosized particles and simultaneously entrap RNA therapeutics. Having this in mind, Lang et al. used peptide self-assembly nanoplatforms to deliver siRNA for the treatment of prostate cancer (Figure 10) [211]. In this study, siRNAs against the cancer-associated fibroblasts (CAFs) were loaded inside a type of cell-penetrating peptide (CPP)-based nanoparticle. This siRNA could specifically downregulate the C–X–C motif chemokine ligand 12 (CXCL12) of CAFs. According to findings, the cell invasion, migration, and angiogenesis of the tumor were considerably inhibited via silencing the expression of CAFs to about 88.7%, leading to a reduction in the prostate tumor size [211].

### 5.4. Cyclodextrin

Cyclodextrins are a family of cyclic oligosaccharides that are extensively applied in the pharmaceutical industry [212]. Although cyclodextrins are excellent solubilizers and stabilizers, their modification is of interest for providing promising nanocarriers to deliver molecules such as proteins and nucleic acids [213]. The first delivery of siRNA in cancer therapy was provided by cyclodextrin-containing polymers [214], while further studies focused on using cyclodextrin-modified nanoparticles in the delivery of siRNA.

**Figure 11:**
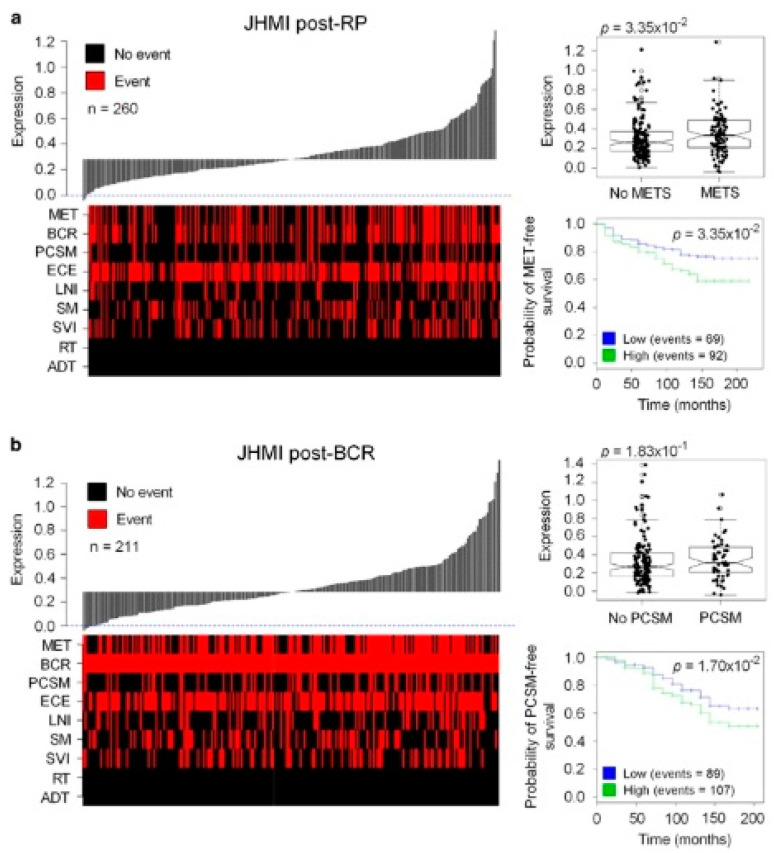
Overexpression of NRP1 as a prognostic of metastatic progression and cancer-specific mortality in cancer patients. Waterfall plots indicating in the overexpression of NRP1 in JHMI patients: (**a**) post-RP and (**b**) post-BCR samples. ECE, extra-capsular extension; LNI, lymph node invasion; MET, metastasis; SM, surgical margin; SVI, seminal vesicle invasion. Boxplots showing *NRP1* expression in patients positive and negative for METS (**a**) and PCSM (**b**). Kaplan–Meier curves indicating MET-free (**a**) and PCSM-free (**b**) survival for *NRP1* high- and low-expression groups. Reprinted with permission from Reference [216].

One instance of use was in the delivery of neuropilin-1 (NRP-1), a transmembrane glycoprotein involved in the induction of angiogenesis via interacting with members of the VEGF family [215]. NRP-1 undergoes upregulation in PCa cells, resulting in proliferation and malignancy (Figure 11) [216,217]. Additionally, zinc finger E-box binding homeobox 1 (ZEB1) is an upstream mediator of EMT and contributes to the metastasis and invasion of cancer cells via the induction of EMT [218,219]. Accumulating data demonstrate that ZEB1 has high expression in PCa cancer cells and is correlated with the progression and metastasis of these cancer cells [220,221]. Cyclodextrin nanoparticles were designed for the delivery of siRNA-ZEB1 and siRNA-NRP-1 in PCa therapy. In order to maximize the targeted delivery and capability of cyclodextrin nanoparticles, their surface was modified with folate to selectively target PCa cells. These nanocarriers are capable of protecting siRNAs against degradation by serum nucleases. The expressions of ZEB1 and NRP-1 showed a decrease with siRNA-ZEB1- and siRNA-NRP-1-loaded cyclodextrin nanoparticles in PCa cells, suggesting the capability of these nanocarriers for the delivery of siRNAs and the effective treatment of prostate cancer via knocking down the level of expression to about 76.99% ± 10.89% [222].

The majority of studies are using cell lines for research, and additional research is required to understand the efficacy and specificity of siRNAs in animal models and eventually humans.

**Figure 12:**
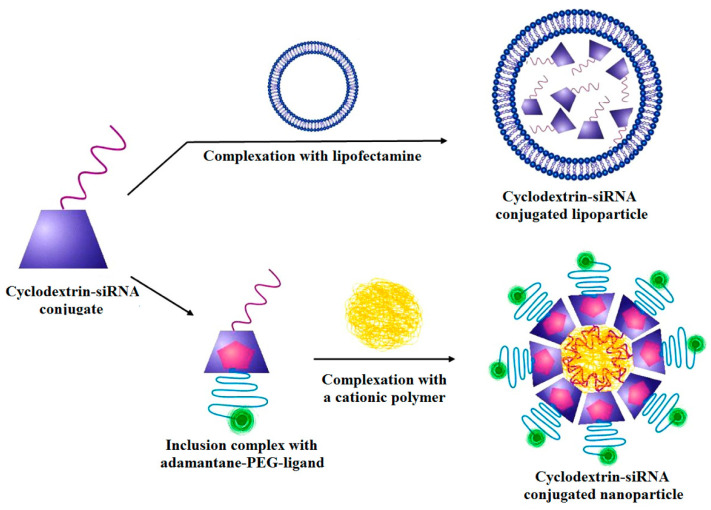
Schematic of lipofectamine–cyclodextrin–siRNA complex and targeted cyclodextrin–siRNA–polymer complex. PEG, polyethylene glycol. Reprinted with permission from Reference [223].

In one study, two types of siRNA (against prostatic cancer cells with overexpression of PLK-1 and luciferase genes) were conjugated to the cyclodextrin to prepare cyclodextrin-based delivery systems, in which the conjugation was done based on applying two types of non-cleavable and cleavable linkers. The as-fabricated conjugates were used in three different forms to obtain to the best system for siRNA delivery, including polycationic cyclodextrin, the complex of cyclodextrins with lipofectamine 2000, and a targeted cyclodextrin–siRNA–polymer complex (which was composed of cationic chitosan in the core covered by siRNA–cyclodextrin and targeted by adamantyl-polyethylene glycol (PEG) ligands). In this study, the effectiveness of cyclodextrin as a delivering agent for the siRNA was confirmed. Moreover, it was observed that the cleavable types of delivery systems showed a higher ability to knock down genes (about 57% expression) in comparison to the non-cleavable ones (about 73% expression). The superior performance was conducted from the targeted formulation which used a receptor-mediated endocytosis method to deliver the siRNA into the cells (Figure 12) [223].

### 5.5. Magnetic Nanoparticles

**Figure 13:**
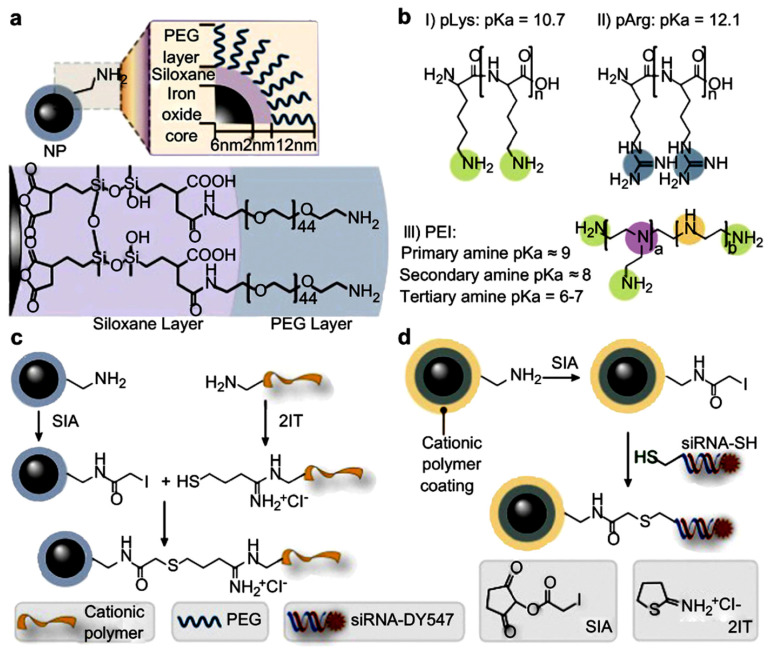
Chemical scheme for synthesis of magnetic nanovectors. (**a**) The amidated PEG-passivated iron oxide NPs used as the base NP for construction of transfection vectors in this study. (**b**) Chemical structures of the cationic polymers used to functionalize the NPs. (**c**) Covalent attachment of cationic polymers to NPs. (**d**) Covalent attachment of Cy5 modified siRNA to NPs. 2IT, 2-iminothiolane; pArg, poly-arginine; PEG, polyethylene glycol; PEI, polyethylenimine; pLys, polylysine; NP, nanoparticle; SIA, succinimidyl iodoacetate. Reprinted with permission from Reference [224].

Magnetic nano-vectors are a class of carriers which were used in a study for the delivery of siRNA to pancreatic cancer cells. These magnetic nano-vectors were fabricated via coating the iron oxide nanoparticles with two polymeric layers of siloxane and polyethylene glycol (PEG) at first, which were then functionalized with positively charged polymers (poly-arginine (pArg), polylysine (pLys), and polyethylenimine (PEI)) that led to the preparation of three different formulations. The siRNA components (labeled by the DY-547 fluorescence tag) against the green fluorescence protein (GFP) transgene cells were loaded on the nano-vector. Different positively charged polymeric layers were used to assess which was more biocompatible and more efficient for siRNA delivering. The result of the study revealed that the pLys-coated nano-formulation was more efficient and safer for siRNA delivery to cancer cells and improved gene silencing ability (about 24%). Indeed, it was observed that this formulation used a different method for cell penetration, allowing escape from lysosomal enzymes, thus enhancing its performance (Figure 13) [224].

**Figure 14:**
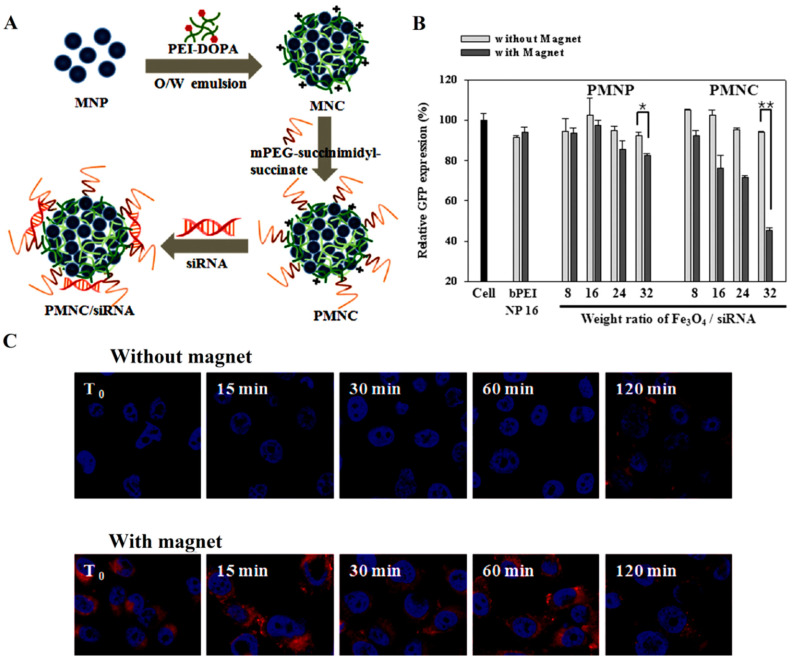
(**A**) Schematic for the polyethylene glycol magnetic nanocluster (PMNC)/siRNA preparation. (**B**) Silencing effect of different concentrations of PMNP and PMNC GFP. (**C**) Effect of magnetic targeting on the transfection of PC-3 cells. Abbreviations: MNC, magnetic nanocluster; MNP, magnetic nanoparticles; O/W, oil in water; GFP, green fluorescence protein; PMNC/siRNA, polyethylene glycol magnetic nanocluster/siRNA; PMNP, polyethylene glycol magnetic nanoparticle. Reprinted with permission from Reference [225].

In another study, magnetic nanoparticles were used as targeting agents for efficient siRNA delivery to the prostate cancer cells. This was done based on a phenomenon known as transfection, in which an external magnetic force was applied to enhance the delivery of genes to the targeted site. To achieve this aim, nanoclusters of oleic acid–magnetic nanoparticles in a polymeric solution of 3,4-dihydroxy-l-phenylalanine (DOPA)-PEI were prepared via an oil-in-water method. Surface functionalization of the nanoparticles was done via applying PEG, which led to stable hydrophilic particles. In the end, siRNA (designed against GFP) was loaded on the nanocluster to attain the final nanosystem. The results of this study showed that nanocarriers containing magnetic nanoparticles (PMNPs) could reduce the silencing of GFP expression by about 18%, while magnetic nanoclusters containing the carrier (PMNCs) showed a 55% reduction in gene expression, in response to the greater amount of magnetic agents present in their structure (Figure 14) [225].

Accumulating data show that functionalized nanoparticles can provide targeted delivery of genes and drugs, with low side effects and partial drug resistance [226,227,228]. As an example, superparamagnetic iron oxide nanoparticles (SPIONs) were proven beneficial in therapeutic and diagnostic imaging [229]. SPIONs can be used for concentrating active agents because they provide enhanced permeability and retention (EPR) [230]. These properties make SPIONs promising candidates in the delivery of genes and drugs and, in this way, they can be used for the delivery of siRNA. A disintegrin and metalloproteinase 10 (ADAM10) is a novel target in cancer therapy [231,232], and it was shown that loading siRNA-ADAM10 on SPIONs enhances their efficacy in reducing the expression of ADAM10, resulting in a decrease in viability and proliferation of PCa cells by about 26% for 10 nM of the complex after 120 h [233].

### 5.6. Gold Nanoparticles

**Figure 15:**
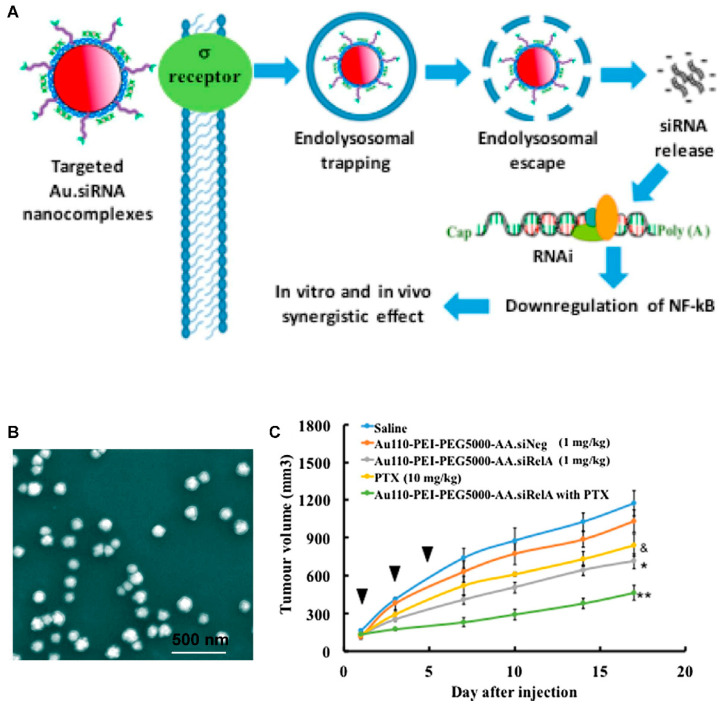
(**A**) Schematic illustration on functionalized gold nanoparticles (NPs) for prostate cancer therapy. (**B**) SEM image of functionalized Au NPs. (**C**) PC-3 xenograft tumor growth reduction following treatment with anti-RelA siRNA (~1 mg/kg) in different formulations (WR40) with or without paclitaxel on days 1, 3, and 5. PTX, paclitaxel; PEG-AA, anisamide-targeted polyethylene glycol. Reprinted with permission from Reference [241].

Gold nanoparticles (NPs) with size- and shape-dependent optical properties generated by surface plasmon resonance (SPR) are extensively applied in biomedicine as contrast agents, photothermal agents, and radiosensitizers [234,235,236,237]. The affinity of gold nanoparticles for biomolecules makes them appropriate options for gene and drug delivery [238]. As an example, the folate receptor is upregulated in PCa cells, and surface modification of nanocarriers with folic acid was shown to enhance the capability of nanoparticles in targeting PCa cells [239]. siRNA-RelA-loaded gold nanoparticles were able to diminish the survival of PCa cells via selective targeting of folate receptors, with diminishment of proliferation and survival of cancer cells resulting from the improved gene silencing (up to 35%) in comparison to control and free siRNA [240]. Functionalization of gold NPs with polymers enhances the drug loading capacity of gold to deliver siRNA. For instance, polyethylenimine (PEI) and PEGylated anisamide, a ligand targeting the sigma receptor, were used to modify the surface of Au NPs (Figure 15). In vivo results showed the sustained release of siRNA was achieved, exhibiting substantial proliferation inhibition (more than 60%) in a PC3 xenograft mouse model without an enhancement in toxicity. This carrier also showed about 40% gene knockdown [241].

Another example is polo-like kinase 1 (PLK1), a member of the serine/threonine protein kinase family, which contributes to a number of biological processes such as mitosis, meiosis, spindle assembly, and centrosome maturation [242,243]. PLK1 is an oncogene and can enhance the malignancy and proliferation of cancer cells [244,245,246]. Multifunctional gold nanorods are able to effectively deliver siRNA-PLK1 to PCa cells and diminish their viability and survival [247]. In previous sections, we demonstrated that the surface modification of nanoparticles by PSMA increases their capability in targeting PC cells. It is worth mentioning that transferrin (Tf) ligands can be implemented for selectively targeting PCa cells, as they are upregulated in PCa cells [248,249,250]. Gold nanoparticles can target the Tf receptors on PCa cells to deliver siRNA to PCa cells, resulting in an inhibition of RelA (up to 35%) and a diminution in the growth and survival of cancer cells [251].

**Figure 16:**
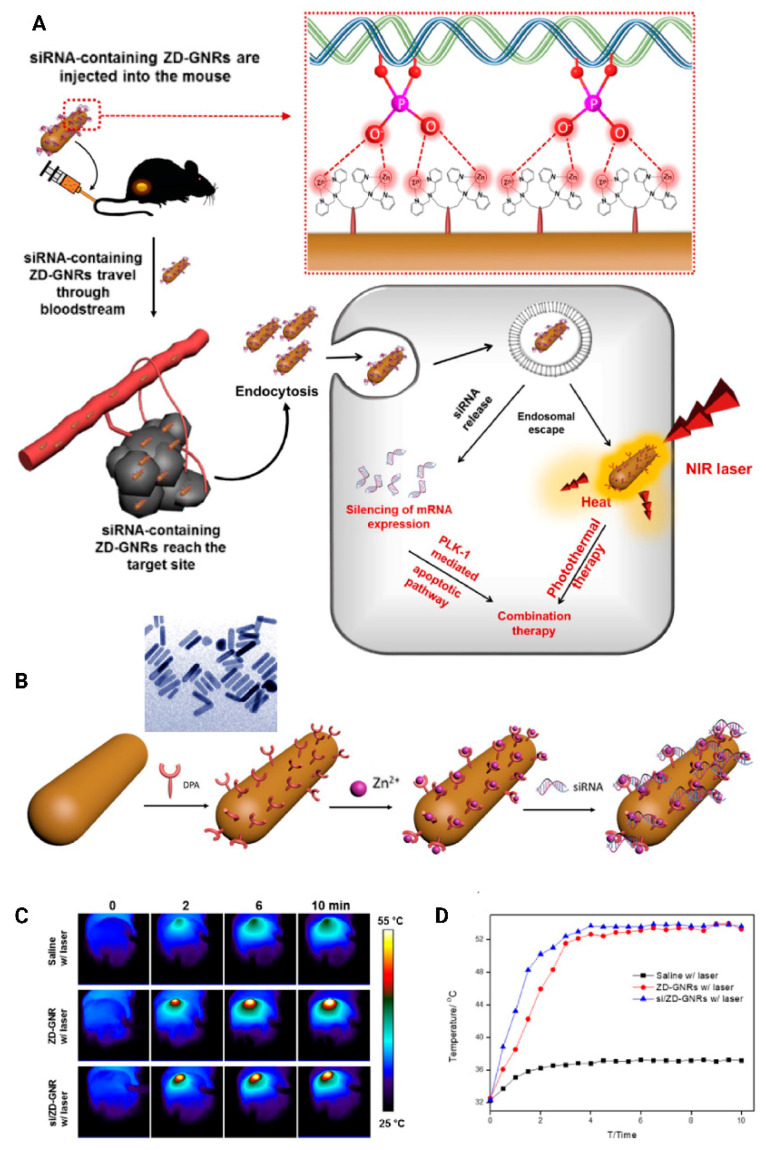
(**A**) Schematic illustration of specific interaction between the Zn (II)–dipicolylamine (Zn-DPA) and phosphate groups of siRNAs and combined anti-PLK1 gene therapy/photothermal therapy upon laser irradiation after the accumulation of siPLK/ZD–gold nanorods (GNRs) at the target tumor tissues. (**B**) Schematic illustration of assembly of siRNA/ZD-GNRs with SEM image of the nanorods. Thermographic images (**C**) and temperature changes of the tumor area (**D**) of the mice treated with saline, ZD-GNRs, and siPLK/ZD-GNRs upon 10 min of laser exposure. Zn-DPA, Zn (II)–dipicolylamine; ZD-GNR, Zn (II)–dipicolylamine–gold nanorod; PLK1, polo-like kinase 1. Reprinted with permission from Reference [247].

Gold nanoparticles were also applied for theranostic applications including bioimaging of genes, as well as delivery and photothermal therapy. In light of this, Au nanorods were used for combination gene therapy (to deliver siRNA) and photothermal therapy along with photoacoustic imaging applications. The nanodevices demonstrated a substantial anticancer effect in a PC-3 tumor mouse model, along with an 85% reduction in the gene expression (Figure 16) [247].

## 6. Conclusions and Remarks

In this review, we evaluated the use, efficacy, and specificity of siRNA in PCa therapy. To date, a high number of genes were targeted by siRNA for the treatment of PCa, including MDM2, IGHG1, VEGF, Neu3, PARP1, and HIF-1α. The goal of targeting these genes using siRNA is to suppress the growth, metastasis, and angiogenesis of PCa cells. Additionally, siRNAs can provide conditions for the enhanced anti-tumor activity of chemotherapeutic agents such as cisplatin. A caveat of siRNA use is its off-target effect. For targeted siRNA delivery, there were efforts to apply siRNAs to tumors using various vehicles, such as dendrimers, magnetic nanoparticles, polymeric nanoparticles, micelles, gold nanoparticles, and nanobubbles. These nanoplatforms considerably enhance the efficacy of siRNA in silencing, as well as its specificity in targeting genes and its half-life, protecting it from degradation. Here, we cite the work of researchers who successfully showed the use of chemotherapeutic agents such as docetaxel co-delivered with siRNAs to provide more effective PCa therapy. Noteworthy, groove modification [261], caging siRNA [262], cholesterol modification for nuclease protection [263] of clinical trials investigating the use of siRNA-loaded nanocarriers is perhaps due to safety concerns. As we described in Section 1, there was a clinical trial using siRNA-loaded nanoparticles with excellent biocompatibility and no toxicity. Furthermore, in vivo and in vitro experiments demonstrated the high efficiency of nanocarriers in the delivery of siRNA in PCa therapy. Thus, these results can be translated into the clinic. Another problem associated with siRNA therapies is the transient effect of siRNAs that need frequent administration. Nanoparticles can provide prolonged release of siRNA, enhancing its efficacy and providing longer gene silencing.

## Figures and Tables

**Table 1:** The efficacy and specificity of siRNAs in targeting signaling pathways in PCa therapy.

Cell Line	Target Gene	Major Outcomes	Refs
PCa cell line PC-3 (androgen-insensitive cells)	*MDM2*	Enhancing cytotoxicity of cisplatin against cancer cells, and induction of caspase-3 and -9	[116]
Human prostate cancer cell lines (PC3, LNCaP)	*IGHG1*	Stimulation of apoptosis and inhibition of proliferation	[117]
DU-145 (human prostate cancer cell line)	*VEGF*	Suppressing proliferation and angiogenesis	[118]
PC-3M, LNcap and DU145 prostate cancer cell lines	*Neu3*	Suppressing migration and metastasis of cancer cells via down-regulation of MMP-2 and MMP-9	[119]
PC3 cells	*PARP1*	Enhancing sensitivity of cancer cells into docetaxel chemotherapy via downregulation of *PARP1* and subsequent inhibition of *EGF/Akt/FOXO1*	[120]
PC3 cells	HIF-1α	Downregulation of *HIF-1α* is corelated with induction of apoptosis and cell-cycle arrest at synthesis (S) and gap 2 (G2)/mitosis (M) phase	[121]
LNCaP cells and LAPC4 cells (androgen-sensitive human PCa cell lines), and C4-2 cells (androgen-independent human PCa cell line)	Androgen receptor (*AR*)	Suppressing metastasis of cancer cells	[122]
Human prostate carcinoma cell lines LNCaP and PC-3	*EGR-1*	Enhancing *p21* activity and stimulation of apoptosis	[123]
PC3 cells	*ADAM17*	Interfering with proliferation and DNA synthesis, and stimulation of cell cycle arrest at S phase	[124]
Human prostate cancer cell LNCaP and its sublines (C4, C42, C4-2B), ARCaP cell lines IA-8, IF-11, and PC-3, DU-145, TSU-PR1	*DNMT3*	Induction of cell-cycle arrest and apoptosis	[125]
Human prostate cell lines PNT2 (benign) and PC-3M_parental_ (highly malignant)	*RPL19*	Impairing proliferation and stimulation of apoptosis	[126]
EnzR-PCa cell lines	*MALAT1*	Sensitizing cancer cells to androgen therapy	[127]
PC-3 and DU145 human prostate cancer cells	*GRP78*	Stimulation of apoptosis and suppressing metastasis	[128]
LNCaP cells	*AR*	Stimulation of apoptosis and sensitizing cancer cells to androgen therapy	[129]
PC3 cells	*JNK-1*	Stimulation of apoptosis, DNA fragmentation, and reducing viability of cancer cells	[130]
RWPE-1, DU145, PC-3, and LNCaP cell lines	*HMGN5*	Triggering mitochondrial-mediated apoptosis via impairing mitochondrial membrane integrity	[131]
Human prostate cancer PC-3 cell lines, which express prostate-specific antigens (PSAs), IGF-1R, and IRS1 (10–12)	*Cytohesin-1*	Downregulation of cytohesin-1 is associated with inhibition of IGFR signaling and desirable prognosis	[132]
PC-3 and LNCaP prostate carcinoma cell lines	*EGR-1*	Triggering apoptosis and inhibition of growth via downregulation of *EGR-1*, and suppressing its downstream targets *NF-κB* and *AP-1*	[133]

**Table 2:** siRNA-loaded nanocarriers with implications in PCa therapy.

Vehicle	Target Gene	In Vitro/In Vivo	Animal Model	Cell Line	Zeta Potential (mV)	Size (nm)	Entrapment Efficiency (EE) (%)	Results	References
Lipid nanoparticle	Androgen receptor (*AR*)	In vitro In vivo	Mice bearing LNCaP tumors	LNCaP and PC-3 human PCa cell lines	-	Up to 84.5	-	Downregulation of androgen receptor and interfering with proliferation	[151]
Peptide dendrimer	*HSP27*	In vitro In vivo	5.0-week-old male BALB/c nude mice bearing PC3 cells	PC3 cells	+18.5 to +22.3	50–70	-	High cellular uptake, effective gene silencing, and reducing proliferation and viability of cancer cells	[170]
Polymeric nanoparticles	*GRP78*	In vitro In vivo	PC-3 prostate cancer-bearing mice	PC3 cells	−23.8 to −24.2	36.4–39.7	82.4	Co-delivery of siRNA-GRP78 and docetaxel, and suppressing invasion and proliferation of cancer cells	[173]
Multifunctional polymeric nanoparticles	*PHB1*	In vitro In vivo	LNCaP tumor-bearing male athymic nude mice	Luc-HeLa and PCa cell lines (LNCaP, PC3, DU145, 22RV1)	+14	56.6	90.6	Downregulation of PHB1, endosomal penetration, and inhibition of proliferation and invasion of PCa cells	[182]
Micelle	*SREBP1*	In vitro In vivo	Mouse model	PC-3 and C4-2B cells	+20.3 to +26.9	100	-	Co-delivery of siRNA-SREBP1 and docetaxel, deep tumor penetration, protection of siRNA, and suppressing cancer malignancy	[190]
Peptide	*EGFP*	In vitro	-	PC3 cells	+25.4	131.5	-	Targeted delivery, high cellular uptake, excellent biocompatibility, and reducing malignancy of cancer cells	[203]
Peptide	*CDCA1*	In vitro In vivo	NOD/SCID mice	Human PCa cell line DU145, PC3, LNCap, and the human prostate epithelial RWPE-1 cells	-	-	-	Downregulation of *CDCA1*, inhibition of mitosis, and induction of apoptotic cell death	[210]
Cyclodextrin conjugate	*PLK1*	In vitro	-	U87 and DU145 cells	-	-	-	Downregulation of *PLK1*, and reducing viability and proliferation of cancer cells	[223]
Magnetic nanoparticles	*ADAM10*	In vitro	-	PC3 cells	−17.9	219.5	-	Downregulation of ADAM10 and induction of apoptosis in cancer cells	[233]
Gold nanoparticles	*RelA*	In vitro	-	LNCaP cells	+46 to +53	113–118	-	High internalization, endo-lysosomal escape, and reducing proliferation and viability of cancer cells	[240]
Multifunctional gold nanorod	*PLK1*	In vitro In vivo	PC-3 xenograft tumor	143B cells	+24.5 to +66.2	48.6–51.13	-	Providing combinational photothermal therapy and gene silencing	[247]
Gold nanoparticle	*RelA*	In vitro	-	PC3 cells	+46	118	-	Downregulation of *RelA*, and suppressing viability and proliferation of cancer cells	[251]
Nanobubble	*FoxM1*	In vitro In vivo	Mice bearing PC3 cells	LNCaP cells	+24.07	479.83	-	Improved transfection efficiency, stimulation of apoptosis and cell-cycle arrest, and reducing tumor growth (in vivo)	[252]
Chitosan nanoparticles	*Snail*	In vitro	-	PC-3 human metastatic prostate cancer cell line	+1.8	169	-	Inhibition of metastasis of cancer cells via upregulation of epithelial markers E-cadherin and Claudin-1	[253]
Cyclodextrin nanoparticles	*ZEB1* *NRP-1*	In vitro	-	PC3 and LNCaP cells	−9.07 to +46.42	Less than 200	-	Downregulation of *ZEB1* and *NRP-1*, inhibition of metastasis, and suppressing angiogenesis	[222]
Polymeric nanoparticle	*VEGF*	In vitro In vivo	PC-3 xenograft tumors	PC3 cells	+1.8	240	-	High cellular uptake through endocytosis, targeted delivery, and downregulation of *VEGF*	[254]
Layer-by-layer nanoparticle	*P44/42 MAPK*	In vitro In vivo	Mouse model	CWR22R cells	+30.5	170–179	56.7	Co-delivery of docetaxel and siRNA-*MAPK*, leading to suppressing invasion and malignancy of cancer cells	[255]
Aptamer chimera	*EGFR* *Survivin*	In vitro In vivo	Mouse model of PCa	Cell lines including PC3, BXPC3 and T-24	-	-	-	Selective targeting of PSMA-overexpressing PCa cells, downregulation of *EGFR* and *survivin*, and stimulation of apoptosis	[256]
Microbubble	*Survivin*	In vitro In vivo	Xenograft mouse tumor model	Human PCa cell lines PC-3 and LNCaP	-	-	-	Co-delivery of siRNA-*survivin* and doxorubicin, and suppressing growth and viability of cancer cells (both in vitro and in vivo experiments)	[257]
Peptide	*Survivin*	In vitro In vivo	LNCaP xenografts in nude mice	PC3 cells	-	-	-	Reducing the viability of cancer cells, and induction of apoptosis	[258]
Gold nanoparticle	*RelA*	In vitro	-	PC3 cells	+27.6	62.8	-	Targeting sigma receptor using anisamide-modified gold nanoparticles, silencing RelA gene, and diminishing viability and survival of cancer cells	[259]
Cyclodextrin	*PLK1*	In vitro	-	DU145 and PC3 cells	+10.28 to +27.8	Less than 300 nm	-	Selective targeting PCa cells by binding into sigma receptors, downregulation of *PLK1* gene, and improving prognosis	[260]

## Abbreviations

PCa	prostate cancer
LHRH	luteinizing hormone releasing hormone
ARs	androgen receptors
mCRPC	metastasis castration-resistant prostate cancer
PSA	prostate-specific antigen
DRE	digital rectal examination
FZD	Frizzled
KRT5	keratin 5
miR	microRNA
lncRNAs	long non-coding RNAs
siRNA	small interfering RNA
RNAi	RNA interference
RISC	RNA-induced silencing complex
mRNA	messenger RNA
PEP	phosphoenopyruvate
NF-κB	nuclear factor kappa B
Bcl-2	B-cell lymphoma 1
SATB1	special AT-rich sequence-binding protein 1
TRIM24	tripartite motif-containing protein 24
CIP2A	cancerous inhibitor of protein phosphatase 2A
PARP1	poly(ADP-ribose) polymerase-1
EMT	epithelial-to-mesenchymal transition
HIF-α	hypoxia-inducible factor-1α
ROS	reactive oxygen species
JNK	c-Jun *N*-terminal kinase
MAPK	mitogen-activated protein kinase
ESM-1	endothelial cell-specific molecule-1
VEGF	vascular endothelial growth factor
SALL4	Sal-like 4
TLRs	Toll-like receptors
RGD	arginine–glycine–aspartic acid
PHB1	prohibitin-1
PSMA	prostate-specific membrane antigen
i.v.	intravenous
SPIONs	superparamagnetic iron oxide nanoparticles
EPR	enhanced permeability and retention
ADAM10	a disintegrin and metalloproteinase 10
GRPR	gastrin-releasing peptide receptor
CDCA1	cell division cycle-associated protein 1
SPR	surface plasmon resonance
PLK1	polo-like kinase 1
Tf	transferrin
ZEB1	zinc finger E-box binding homeobox 1
DANCR	differentiation antagonizing non-protein coding RNA
MEG3	lncRNA maternally expressed gene 3
PCA3	prostate cancer antigen 3
DRAIC	downregulated RNA in cancer
PCAT29	prostate cancer-associated transcript 29
GAS5	growth arrest-specific 5
CTBP1-AS	C-terminal binding protein 1 antisense
PCGEM	prostate cancer gene expression marker 1
MALAT-1	metastasis-associated lung adenocarcinoma transcript 1
NEAT1	nuclear-enriched abundant transcript 1
PCAT5	prostate cancer-associated transcript 5
SChLAP1	second chromosome locus associated with prostate 1
HOTAIR	HOX transcript antisense RNA
SOCS2-AS1	cytokine signaling 2 antisense transcript 1
TIMP 2/3	tissue inhibitor of metalloproteinase
EZH2	enhancer of zeste homolog
ZNF217	zinc finger protein 217
ZEB1	zinc-finger E-box binding homeobox 1
PRUNE2	prune homolog 2
NKX3-1	homeobox protein Nkx 3.1
FOXA1	forkhead box protein A1
BCL4	B-cell lymphoma like-2 like protein 4
SMAD3	mothers against decapentaplegic homolog 3
CTBP1	C-terminal binding protein 1 antisense
HDAC-Sin3A	histone decarboxylase paired amphipathic helix protein Sin3a complex
TMEM48	transmembrane protein 48
CKS2	cyclin-dependent kinase regulatory subunit 2
hnRNP A1	heterogeneous nuclear ribonucleoprotein A1
U2AF65	U2 small nuclear RNA auxiliary factor 2
DAB2IP	disabled homolog 2-interacting protein
TMPRSS2	transmembrane protease, serine 2
ERG	ETS (E-twenty-six)-related gene
SWI/SFN complex	switch/sucrose non-fermentable complex
TNSF10	tumor necrosis factor superfamily member 10
MDM2	mouse double minute 2 homolog
